# The wooly mutation (*wly*) on mouse chromosome 11 is associated with a genetic defect in *Fam83g*

**DOI:** 10.1186/1756-0500-6-189

**Published:** 2013-05-09

**Authors:** Legairre A Radden, Kevin M Child, Elisabeth B Adkins, Damek V Spacek, Aaron M Feliciano, Thomas R King

**Affiliations:** 1 Biomolecular Sciences, Central Connecticut State University, 1615 Stanley Street, New Britain, CT 06053, USA

**Keywords:** Mouse model, Hair variant, Positional candidate approach, *Slc5a10*, *Fam83g*

## Abstract

**Background:**

Mice homozygous for the spontaneous wooly mutation (abbreviated *wly*) are recognized as early as 3–4 weeks of age by the rough or matted appearance of their coats. Previous genetic analysis has placed *wly* in a 5.9 Mb interval on Chromosome 11 that contains over 200 known genes. Assignment of *wly* to one of these genes is needed in order to provide probes that would ultimately facilitate a complete molecular analysis of that gene’s role in the normal and disrupted development of the mammalian integument.

**Results:**

Here, a large intraspecific backcross family was used to genetically map *wly* to a smaller (0.8 Mb) span on mouse Chromosome 11 that includes fewer than 20 genes. DNA sequencing of the coding regions in two of these candidates known to be expressed in skin has revealed a 955 bp, *wly*-specific deletion. This deletion, which lies within the coordinates of both *Slc5a10* [for solute carrier family 5 (sodium/glucose cotransporter), member 10] and *Fam83g* (for family with sequence similarity 83, member G), alters the splicing of mutant *Fam83g* transcripts only, and is predicted to result in a severely truncated (probably non-functional) protein product.

**Conclusion:**

We suggest that this mutation in *Fam83g* is the likely basis of the mouse wooly phenotype.

## Background

**Figure 1:**
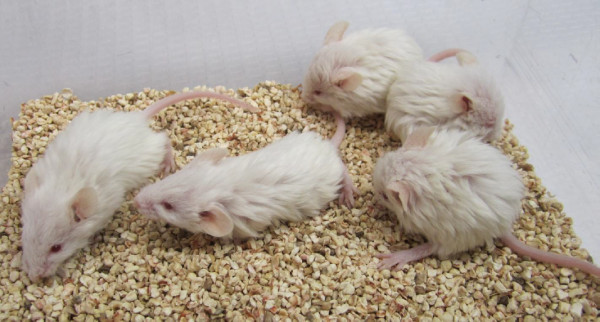
**Three-week old mice, homozygous for the wooly (*wly*) mutation.**

The spontaneous wooly (or woolly) mutation (abbreviated *wly*) was initially identified at The Jackson Laboratory (Bar Harbor, ME, USA) among a litter of inbred NOD/ShiLtJ mice [1]. Mutants are recognized as early as 3–4 weeks of age by the rough or matted appearance of their coats (see Figure 1), but—in spite of this presentation—all hair types examined (auchene, guard, zigzag and vibrissae) appear microscopically normal [1], and histological examination of skin has revealed no marked anomaly compared to normal-coated (heterozygous) littermates [1]. When *wly* was mapped to mouse Chromosome (Chr) 11, it was immediately tested for genetic complementation in crosses with the waved 2 (*wa2*) mutant [2], but since no affected progeny were produced, these recessive variants must be due to defects in distinct, albeit syntenic, genes [1]. Indeed, homozygosity mapping based on 54 affected F_2_ animals has placed *wly* between *D11Mit313* and *D11Mit261*[1], a 5.9 Mb interval that contains over 200 known genes [3], but does not include *wa2* or any other obvious gene candidates.

To associate this mutation with a causative molecular defect, *wly* was fine-mapped to a genetic region where fewer than 20 genes are located—only a few of which are known to be expressed in skin. Direct sequence analysis of the coding regions in two of these candidates has identified a mutant-specific defect in *Fam83g* (for family with sequence similarity 83, member G) that we propose to be the likely genetic basis of the *wly* mutation.

## Methods

### Mice

Standard inbred strains A/J, C57BL/6J, and NOD/ShiLtJ; and NOD/ShiLtJ-*wly*/J mutant mice were obtained from The Jackson Laboratory (Bar Harbor, ME, USA). Mice homozygous for the mutant *wly* allele were reliably identified by the matted appearance of their coats which is first evident by 3–4 weeks of age and persists throughout life. Both male and female *wly/wly* homozygotes appear to be fully fertile, and we have maintained the NOD/ShiLtJ-*wly*/J line since 2009 by crossing homozygotes. The treatment and use of all mice in this study were compliant with protocols approved by the Institutional Animal Care and Use Committee (IACUC) at Central Connecticut State University (New Britain, CT, USA).

### DNA analysis

Genomic DNA was isolated from 2–4 mm tail-tip biopsies taken from two-week-old mice, using Nucleospin® kits from BD Biosciences (Palo Alto, CA, USA), as directed. DNA samples from standard inbred and mutant strains that we do not routinely maintain in our colony were purchased from The Jackson Laboratory’s Mouse DNA Resource.

The polymerase chain reaction (PCR) was performed in 13 ul reactions using the Titanium® PCR kit from BD Biosciences, as directed. Oligonucleotide primers for PCR were designed and synthesized by Invitrogen (Carlsbad, CA, USA), based on sequence information available online [3,4]. In addition to standard microsatellite markers [5] on Chr 11, six DNA markers based on single-nucleotide-polymorphisms previously reported to differ between strains A/J and NOD/ShiLtJ [3,4] were also scored. These markers (herein designated *SNP1-6*) are described in detail in Additional file 1 & Additional file 2. To visualize PCR product sizes, reactions plus 2 ul loading buffer (bromophenol blue in 20% Tris-buffered sucrose) were electrophoresed through 3.5% NuSieve® agarose (Lonza, Rockland, ME, USA) gels. Gels were stained with ethidium bromide (0.5ug/mL) and photographed under ultraviolet light. For sequence analysis, about 1.5 ug of individual PCR amplimers were purified and concentrated into a 30 ul volume using QIAquick® PCR Purification kits (Qiagen, Valencia, CA, USA). Amplimers were shipped to SeqWright, Inc. (Houston, TX, USA) for primer-extension sequencing.

### mRNA analysis

Total RNA was isolated from tail skin samples taken from 3-month-old mice using the Nucleospin® RNA L kit by Macherey-Nagel (Easton, PA, USA). cDNA was generated using the SMARTer™ RACE cDNA amplification kit (Clontech Laboratories). To amplify *Slc5a10*-specific cDNA, primers *1F* (5’ TGTTCCGGGACCCTTCCACAGGAGACCT 3’), taken from Exon 9, and *1R* (5’ ATGACCAGCCGTCCCACCAGCAGCAACT 3’), taken from Exons12 and 13, were used in a “step-down” PCR reaction. The products of this initial reaction were diluted 1:10 in Tricine-KOH buffer (10 mM, pH 8.5) plus 1 mM EDTA, and were amplified again in a standard PCR reaction using a nested primer pair: *2F* (5’ AGCGGTCCCTGTCTGCCCGGAACTTGAA 3’), taken from Exon 10, and *2R* (5’ TGGGCATCAGCTCCATGACCAGCTTCGGGT 3’), taken from Exon 11. To amplify *Fam83g*-specific cDNA, primers *3F* (5’ ACGGGCAGCCGCACATCAAGGAAGTGGT 3’), taken from Exon 1, and *3R* (5’ AGCACAATGGGCTCTGGCTCTGGCTCCT 3’), taken from Exon 4, were used in a standard PCR reaction. The products of this initial reaction were diluted (as above), and were amplified again with a nested primer pair: *4F* (5’ TGCGCAAGATGGTCAGCCAGGCGCAGAA 3’), taken from Exon 1, and *4R* (5’ ATGGGCTCTGGCTCTGGCTCCTTCTCCA 3’), taken from Exon 4. Final (second-round) amplimers were purified (as described above) and shipped to SeqWright, Inc., for primer-extension sequencing.

## Results

### Meiotic fine-mapping of *wly*

**Figure 2:**
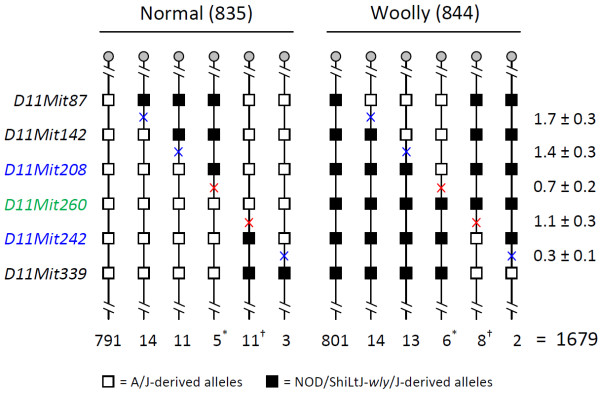
**Segregation of *wly *and six DNA markers on mouse Chr 11 among 1,679 backcross progeny.** Microsatellite markers typed are shown to the left of the diagram. The haplotype depicted is that transmitted by the heterozygous F_1_ dam. Open boxes indicate A/J-derived alleles; filled boxes indicate NOD/ShiLtJ-*wly*/J-derived alleles. The number of progeny that inherited each haplotype is shown below it. The percentage recombination in each marker interval is shown to the right (± 1 standard error). Red crosses represent crossovers in the interval between *D11Mit208* and *D11Mit242*, a span that includes *wly* (since *wly* must lie telomeric to the 11 crossovers marked with an asterisk, and centromeric of the 19 crossovers marked with a dagger). Marker *D11Mit260* (shown in green) did not recombine with *wly* in the backcross panel.

To more precisely locate *wly* on proximal Chr 11, (A/J x NOD/ShiLtJ-*wly*/J)F_1_, *wly/+* females were bred back to NOD/ShiLtJ-*wly/wly* males, producing a large family of 1,679 backcross progeny that segregated for *wly* (and numerous other molecular markers). These progeny were typed for *wly* and six, PCR-scorable, microsatellite markers on Chr 11, as summarized in Figure 2. These data are in agreement with the 1 wild type : 1 mutant ratio expected for a testcross (*χ*^2^ < 0.05; P > 0.8), suggesting that *wly/wly* mice are fully viable, at least compared to heterozygous *wly*/+ littermates. Furthermore, this analysis indicated that *wly* is located between *D11Mit208* and *D11Mit242*, a region of about 4.8 Mb and fully consistent with *wly*’s previously-defined mapping interval between *D11Mit313* and *D11Mit216*[1].

**Figure 3:**
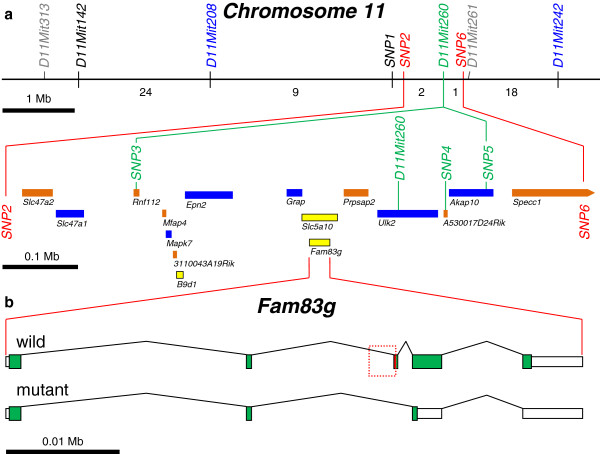
**Physical maps of the *wly *mutation on mouse Chr 11.** (**a**) Molecular markers and genes on mouse Chr 11 that are linked with *wly*. Markers in grey (*D11Mit313* and *D11Mit261*) have been reported by others to flank the *wly* mutation [1]. Segregation data from the large, 1,679-member backcross (shown in Figure 2) place *wly* between the markers shown in blue (*D11Mit208* and *D11Mit242*). Single-nucleotide polymorphisms (*SNP1*-*6*, see Additional file 1 & Additional file 2) were used to further localize crossovers among those backcross mice recombinant between *D11Mit208* and *D11Mit242* (the numbers of crossovers located in each interval are shown below the chromosome). Three such recombinants located *wly* between *SNP2* and *SNP6* (shown in red). A 1-Mb scale bar is shown below the linear arrangement of these markers. An expanded physical map of the markers and genes (represented by colored rectangles) located between *SNP2* and *SNP6* is shown below the chromosome and above a 0.1 Mb scale bar. Markers shown in green were never separated from *wly* in the backcross panel. Mice homozygous for null-alleles of the genes shown in blue have normal furry coats, making these genes poor candidates for being the genetic basis of *wly*. For genes shown in orange, available expression and functional data [3,4] did not overtly implicate skin, but genes shown in yellow are known to be expressed in mouse skin. (**b**) The *Fam83g* gene has been expanded (note the .01 Mb scale bar) to show the arrangement of its five exons. Taller green boxes represent coding regions, and shorter white boxes represent untranslated regions. The portion of Intron 2–3 and Exon 3 boxed in red on the wild type allele is deleted in mutant mice (see also Figure 4a). This disruption is predicted to alter mRNA splicing of the mutant *Fam83g* transcript, as indicated.

To further restrict the physical position of *wly* on Chr 11, mice with a meiotic crossover between *D11Mit208* and *D11Mit242* were typed for six, single-nucleotide polymorphisms (herein designated *SNP1-6*) known to lie in this “critical region”. This analysis identified two recombinants that carried a crossover between *SNP2* and *wly*, and one with a crossover between *wly* and *SNP6*. (No crossovers separated *wly* from *D11Mit260*, or from *SNP3*, *4*, or *5*.) Thus, *wly* must be located between *SNP2* and *SNP6*, an interval that measures less than 0.8 Mb and includes fewer than 20 genes or predicted genes (see Figure 3a).

### Sequence analysis of gene candidates from the *wly*-critical interval

For some of the genes located in the *SNP2* to *SNP6* interval (including *Slc47a1*[6], *Epn2*[7], *Grap*[8], *Ulk2*[9], *Mapk7*[10], and *Akap10*[11]) loss-of-function alleles have been engineered (by others), and null-allele homozygotes have been reported to display no apparent changes to hair development or coat texture, making them unlikely candidates for the gene responsible for the *wly* mutation. For several others (including four predicted genes not shown in Figure 3a), available expression data [3,4] failed to suggest any obvious functional role in skin. By contrast, two other genes in the critical interval, the overlapping *Slc5a10* [for solute carrier family 5 (sodium/glucose cotransporter), member 10] and *Fam83g* (for family with sequence similarity 83, member G) genes, were isolated from cDNA libraries derived from mouse skin [12]. To investigate these “primary” candidates as the possible basis of the mutant wooly phenotype, the coding regions of both genes were sequenced in genomic DNA from A/J, C57BL/6J, NOD/ShiLtJ, and mutant NOD/ShiLtJ-*wly*/J mice.

While this analysis revealed several sites that are polymorphic among these four strains—some new and some previously reported (as summarized in Additional file 3 and shown in detail in Additional file 4 & Additional file 5)—the only sequence distinction found between the coisogenic NOD/ShiLtJ and NOD/ShiLtJ-*wly*/J strains was a 955 bp deletion in the mutant strain (see Additional file 6a) that lies in Intron 10–11 of *Slc5a10* and removes part of Intron 2–3 and Exon 3 of *Fam83g* (see Figure 3b). Because none of 17 other inbred strains tested (including nine strains of Swiss origin, and therefore related to or derived from the NOD strain) show this sequence alteration, while *wly* mutants tested from three distinct colonies do (Additional file 6b), we suggest that this 955 bp deletion is specifically associated with the *wly* mutation.

### A *Fam83g* deletion is likely to be the molecular basis of the mutant wooly phenotype

Because this deletion lies in Intron 10–11 of the *Slc5a10* gene, we amplified sequences between Exons 9 and 13 from cDNA templates based on total RNA isolated from *wly*/*wly* or wild type skin to determine if this mutation could affect splicing of *Slc5a10* transcripts. Amplimers copied from *wly*/*wly* and wild type NOD/ShiLtJ cDNA templates were identical in length, and sequencing verified that Exons 10 and 11 of *Slc5a10* are spliced normally in *wly*/*wly* mutants (data not shown).

**Figure 4:**
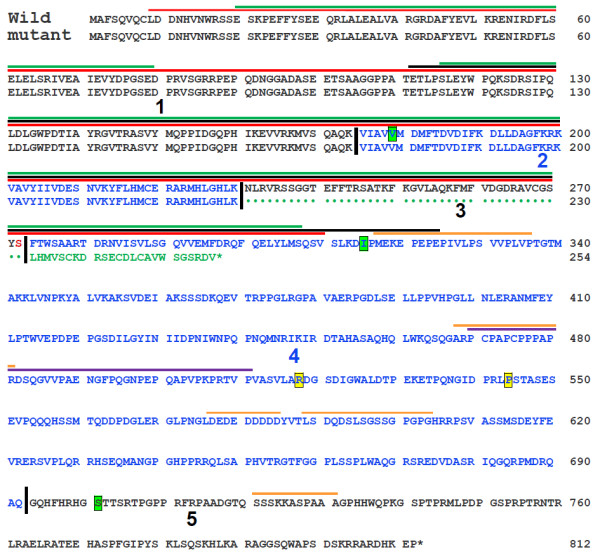
**The predicted amino acid sequence encoded by wild type and mutant alleles of *Fam83g*.** The wild type amino acid sequence is based on our DNA sequence analysis of the C57BL/6J, A/J, and NOD/ShiLtJ inbred strains of mice. The five coding sequence differences we found among these three strains and the NOD/ShiLtJ-*wly* strain are highlighted in green (for three silent, third-position changes) or in yellow (for two nonsynonymous substitutions). All of these polymorphisms are described in detail in Additional file 5. Vertical bars indicate boundaries between odd numbered exons (shown in black) and even-numbered exons (shown in blue). The codon specifying Serine 272 (shown in red) spans Exons 2 and 3. In mutant *Fam83g* mRNA, Exon 3 is skipped (see Figures 3b & Additional file 6c), predicting that Exon 4 sequences will be translated out-of-frame, yielding 24 novel amino acids (shown in green) before an out-of-frame stop codon (*) is encountered, yielding a severely truncated product of only 524 amino acids (*vs.* the normal 812 amino acids). Conserved domains that have been predicted for the wild type Fam83g protein are indicated by horizontal lines on the diagram: red is the domain of unknown function DUF1669, accession number PF07894 [20,21]; black is the phospholipase D/nuclease superfamily domain, accession number SSF56024 [22,23]; green is the N-terminal phospholipase D-like domain, accession number cd09187 [24,25]; purple is the proline rich function unknown, accession number PS50099 [28,29]; and are discussed in the text. Horizontal lines drawn in orange indicate regions that display low complexity [3].

Because this deletion removes the splice acceptor site at the 5’ end of Exon 3 in *Fam83g*, it was predicted that the mutant transcript would likely be spliced such that Exon 2 is joined with Exon 4, rather than Exon 3 (see Figure 3b). This prediction was tested by PCR amplification of sequences between Exon 1 and Exon 4 from cDNA templates based on total RNA isolated from mutant or wild type skin. Sequencing of the 359 bp, *wly*-specific product and the 484 bp, wild type product (see Additional file 6c) demonstrated that the predicted, aberrant Exon 2–4 splice does occur in *wly*/*wly* mutants; while the expected Exon 1-2-3-4 joining occurs in wild type mice. Because the skipped Exon 3 contains 125 nucleotides, the inappropriate junction of Exon 2 with 4 causes a frameshift after Codon 224 that is predicted to introduce 24 novel amino acids in the mutant *Fam83g* gene product before terminating translation quite early in Exon 4 (see Figure 4). Such a severe truncation in the *Fam83g* protein sequence (from an expected 812 a.a. for the normal protein to only 524 a.a. in the mutant) is very likely to negatively impact protein function, and suggests further that this mutation could be the basis of the mutant wooly phenotype.

## Discussion

The eight members of the Fam83 family are mostly uncharacterized proteins, in both mouse and man. Fam83a, also known as tumor antigen BJ-TSA-9 [13], is a novel, tumor-specific protein highly expressed in human lung adenocarcinoma cells. Fam83d, also referred to as spindle protein CHICA [14], is a cell-cycle-regulated spindle component which localizes to the mitotic spindle and is both up regulated and phosphorylated during mitosis. Defects in the gene encoding Fam83h cause autosomal dominant hypocalicified ameliogenesis imperfecta (ADHCAI) [15-19]. Fam83b, c, f, and g are uncharacterized proteins present across vertebrates, while Fam83e is an uncharacterized protein found only in mammals. Some proteins with structural similarity to Fam83g’s N-terminal domain of unknown function (Pfam domain DUF1669, [20,21]; superfamily domain SSF5624, [22,23]; conserved domain cd 09119, [24,25]; see Figure 4) are known to be phospholipases, but this domain in Fam83g shows only trace similarity to the phospholipase D catalytic domain and lacks the functionally-important histidine residue [26,27], so while Fam83g may share a similar 3-dimensional fold with some phospholipase D-like enzymes, it is unlikely to display phospholipase D-like activity. An additional proline-rich domain has been identified from amino acid 470 to 511 (Prosite profiles, PS5099, [28,29]; see Figure 4), but again no function has yet been assigned. Whatever their functional significance, both of these conserved protein domains would be partially or entirely removed by the *Fam83g* mutation in wooly mice (see Figure 4). We predict that this mutant Fam83g protein is, therefore, likely to be non-functional, consistent with our suggestion that the lack of normal Fam83g product in *wly/wly* homozygotes may be responsible for the mutant wooly phenotype.

More direct evidence for a causal link between this deletion and the wooly phenotype would require, for example, transgenic rescue of mutant homozygotes, or recreation of the wooly phenotype in engineered *Fam83g* “knock-out” homozygotes. While such single-addition and single-subtraction experiments are beyond our laboratory’s ability to perform, we anticipate testing for complementation between NOD/ShiLtJ- *wly*/J and a recessive, *Fam83g* knock-out variant (as soon as one becomes available). Non-complementation (*i.e.*, production of phenotypically mutant *wly*/*Fam83g*^*k*.*o.*^ offspring) would provide definitive proof that homozygousity for *Fam83g* defects, alone, is the molecular basis of the mutant wooly phenotype.

No other similar mutations affecting skin or hair have been described in this region on mouse Chr 11 [3], nor are we aware of any human conditions involving skin or hair in the orthologous region of human Chr 17p11-12 [30]. In man, the term “woolly hair” (WH) is used to describe a group of inherited hair shaft disorders characterized by fine and tightly curled hair [31], but—based on their distinct phenotypes, chromosomal locations, and, in some cases, known molecular bases [see [32,33]—none of these described disorders appears related to mouse wooly. We therefore suggest that the NOD/ShiLtJ- *wly*/J mouse strain may provide a unique animal resource, the study of which will be crucial to any future investigation of *Fam83g* and its functional role in the normal or disrupted development of the mammalian integument. For example, it will be interesting to learn where *Fam83g* is expressed in normal and mutant hair follicles (*e.g.*, in the dermal papilla, the epithelial lineage, or other hair-follicle-associated structures), and whether its expression varies during the anagen, catagen and telogen phases of the hair follicle growth cycle. While one previous histological evaluation failed to uncover a microscopic manifestation of the wooly phenotype [1], we hope—especially with molecular probes made possible by the likely genetic assignment of *wly* to *Fam83g*—that *in situ*-based expression or immunohistological analyses can now be approached to finally reveal the cellular basis of the wooly phenotype.

## Conclusion

The 955 bp deletion we describe appears to be specifically associated with the *wly* mutation; it alters the splicing of mutant *Fam83g* transcripts; and is predicted to generate a severely truncated, mutant Fam83g protein. We therefore suggest that this defect is likely to be the molecular basis of the mutant wooly phenotype.

## Competing interests

The authors declare that they have no competing interests.

## Authors’ contributions

LAR and KMC led all aspects of this study, including experimental design, data acquisition and interpretation. DVS and EBA made substantial contributions to the genetic analysis. EBA and AMF participated in the cDNA analysis. TRK conceived of the study, carried out all procedures involving mice, and drafted the manuscript. All authors read, edited, and approved the final manuscript.

## Authors’ information

TRK is a professor in the Biomolecular Sciences Department at Central Connecticut State University (New Britain, CT). LAR, KMC, EBA, DVS, and AMF were undergraduates majoring in Biomolecular Sciences or Biochemistry when they conducted this research.

## Supplementary Material

Additional file 1 **Description of SNP markers referred to in the Radden *et al.* (2013) text.** Click here for file

Additional file 2 **Location of SNP markers referred to in the Radden *et al. *(2013) text.** Click here for file

Additional file 3 **DNA re-sequencing analysis of the exonic portions of the overlapping *Slc5a10 *and *Fam83g *genes in mouse.** Click here for file

Additional file 4 **Description of polymorphisms encountered while re-sequencing *Slc5a10 *in A/J, C57BL/6J, NOD/ShiLtJ, and NOD/ShiLt-*wly*/J mouse DNA.** Click here for file

Additional file 5 **Description of polymorphisms encountered while re-sequencing *Fam83g *in A/J, C57BL/6J, NOD/ShiLtJ, and NOD/ShiLt-*wly*/J mouse DNA.** Click here for file

Additional file 6 **A *Fam83g *deletion specific to the NOD/ShiLtJ-wly/J strain alters mRNA splicing.** Click here for file
