# Supplementary material for: The wooly mutation (wly) on mouse chromosome 11 is associated with a genetic defect in Fam83g
Source: BMC Res Notes. 2013 May 9;6:189. doi: 10.1186/1756-0500-6-189 (PMC3663780; doi:10.1186/1756-0500-6-189)
Supplement: Additional file 1 — Description of SNP markers referred to in the Radden et al. (2013) text. [file 1756-0500-6-189-S1.docx]

**Additional file 1.** Description of SNP markers referred to in the Radden *et al*. (2013) text.

| Designation in Radden *et al.* | Official Designation | Informal Designation | Forward Primer (5’-3’) | Reverse Primer (5’-3’) | Amplimer Length | Alleles (A/B/N/W) | Amplimer Sequence |
| --- | --- | --- | --- | --- | --- | --- | --- |
| *SNP1* | *rs28233795*,  *rs28233794*,  *rs28233793*,  *rs28233792*,  *rs28233791*,  none,  *rs28233790*,  *rs47323643*,  *rs28233789* | SNP D | aaaccagaggccactttgtg | gtacgcactgttgggaggat | 301 | A/G/G/G,  G/A/A/A,  T/C/C/C,  G/A/A/A,  G/A/A/A,  GGGGGG/•/•/•  G/A/A/A,  G/A/A/A,  A/G/G/G | 5' aaaccagagg ccactttgtg TCTCTAGTAG GACCAGAGGG ACTTGGGATT CCCAAAGTAT CCATGGCGTG GCAAG**R**CACT CAGGTCTACA GAGAGACACA C**R**GGTATTGC CCTGGGTGAC TTGTTC**Y**CCA GAAAC**R**TCGA CATTAAGTAT CAAGTCCGCT AAAGGACATG GGAGAGAACC AC**R**CAACAAC CTTCAGAACC TCCAGGGGTA CGCCCGGGGG*CGGTGCTGCT CAGGCAGTTC TA**R**CATTAGT TCCTTCCACA GTGGCTGA**R**C ACTGAGCTCC Catcctccca acagtgcgta c 3' |
| *SNP2* | *rs26970024* | SNP F | tgctggaattcttggcttct | ctagattggcctgtgggtgt | 240 | G/G/T/T | 5’ tgctggaatt cttggcttct CTGGGGCCTA AAGCTTTAAC TGGAGCCCAG AGTGTCTCCT CTGCTTGGGT TTCCCCCAAT TCTCTGCCTC CCTGTCTTGC CTCACCTGCT CTCCTAATGT TCTAAAAGCT ACACATAGAA AACCCACAGT TAACATCATA CGGTCTCACA CTCAGACCCA GGGAACAGTG CTGCCT**K**CCC GTAGCCAGTC CTGCACAGAC acacccacag gccaatctag 3’ |
| *SNP3* | *rs26941299* | SNP E | aggtatcaggcggtcctttt | ggacacacccgtctgcttat | 196 | A/A/G/G | 5’ aggtatcagg cggtcctttt CTACATCCCC AGAAACTCAG CTCCCCTCCC AGAGTCAGCC TGGGGCTCTC ATTCTGCAAC CTCCTGCCAG CCCTGCCCTC ACCAGGCTTG GTAAGCCACT GAGCAAGTGG TCCAA**R**AGGA AGGACTTTCC TGAGTGCTGC TCTCCCAGGA CAGCCAataa gcagacgggt gtgtcc 3’ |
| *SNP4* | *rs26955054* | SNP A | gagtctggcccaatgtgtct | gtgcagttccaagcttctcc | 282 | A/A/G/G | 5’ gagtctggcc caatgtgtct TGTCATAGTA GAACTGTCCT CCTCACCCTT GACTGACCCT GAGGGTGTGT TTGTAATCCT CACCATGGAA CCACTTGCTT ATTGCTTTTG AAATAGCTCT TTGAAAGGCT GGTTTGCAAA TGTACTCTTG TACTAGTGAG GGGATGTTTC CCTGGAATAA TGAGAAATCT CTCTGTCCTG CTTTTATAGA AAAACAAAAC AAAACAAAAC AAAAAAGCAC CCACTCCC**R**A TTCGTTCAAG TAggagaagc ttggaactgc ac 3’ |
| *SNP5* | *rs26954885* | SNP G1 | gccattcggatagtcccata | tggaagagcagcccatactc | 187 | A/A/G/G | 5’ gccattcgga tagtcccata CTGTAACTAC TCTAAGTTCT GCTGACCTCA AAGGTCTCTG TTTATACTAT CAAAAATACA CATATGTCTG TGTTACAATA ACACCTTGTT TCAAAAGCTG GTGGTGCACT GCACTGCACT GCACT**R**TTGA GATGGCTTAG AGGTCAAgag tatgggctgc tcttcca 3’ |
| *SNP6* | *rs26941842*,  *rs26941841*,  none,  *rs26941840* | SNP B | tgaggctggcttctttcatt | cctgtcagccctccatgtat | 368 | A/A/G/G,  A/A/G/G,  G/G/Δ/Δ,  C/C/T/T | 5’ tgaggctggc ttctttcatt CCATAAGATG TCCTCACCAG TATCCATGTT GCCACATGTG CCAGCATTTC CTGTTTTTTT AAAGCCAGAT TTTATTGTGG TGTATGTTCT TACCACATCC CCTC**R**CTTTC TTCCTCTTGG CTGTTATGAA TTGC**R**CTGCT GTGGTCCTAT CTCCTGGCAG CTTATATGAT CCACATGGGG TCA**G**ATTGTT GCTTATGGTA GTAGCAATGG GACCCTGAGG GATGGGATGT GTGTATGCAT GGCCAGGAAG GTCCTGTGGC CATGTAATGG ATGA**Y**TGAGA ACTGGTTCTC ATGTACTTGG GGAGCTCATG GAGATGACGC CACACATCat acatggaggg ctgacagg 3’ |

Primers listed (in lower case) were used to amplify genomic DNA from strains A/J (abbreviated A), C57BL/6J (abbreviated B), NOD/ShiLtJ (abbreviated N), and NOD/ShiLtJ-*wly*/J (designated W). Amplimers were sequenced by primer extension (SeqWright, Inc.; Houston Texas). Nucleotides that differ between two strains are shown in red, where M = A or C, S = C or G, R = A or G, and Y = C or T. Δ denotes a deleted base. An asterisk denotes an insertion of GGGGGG in the A/J strain in *SNP1*. Official designations are from dbSNP Build 132. These data accessed through the Mouse Genome Database at the Mouse Genome Informatics website, The Jackson Laboratory, Bar Harbor Maine. World Wide Web (URL: <http://www.informatics.jax.org> (Accessed September, 2012) and the ensembl Mouse Genome Database, release 64 <http://ensembl.org/Mus_musculus> (Accessed September, 2012).
