# Supplementary material for: The wooly mutation (wly) on mouse chromosome 11 is associated with a genetic defect in Fam83g
Source: BMC Res Notes. 2013 May 9;6:189. doi: 10.1186/1756-0500-6-189 (PMC3663780; doi:10.1186/1756-0500-6-189)
Supplement: Additional file 2 — Location of SNP markers referred to in the Radden et al. (2013) text. [file 1756-0500-6-189-S2.docx]

**Additional file 2.** Location of SNP markers referred to in the Radden *et al*. (2013) text.

| Designation in Radden *et al.* | Official Designation | Informal Designation | Position  (NCBI Build 37) | 5’ Flanking Gene | 3’ Flanking Gene |
| --- | --- | --- | --- | --- | --- |
| *SNP1* | *rs28233795*,  *rs28233794*,  *rs28233793*,  *rs28233792*,  *rs28233791*,  none,  *rs28233790*,  *rs47323643*,  *rs28233789* | SNP D | 11: 60903272,  11: 60903298,  11: 60903323,  11: 60903332,  11: 60903379,  11: 60903416,  11: 60903439,  11: 60903441,  11: 60903465 | *Kcnj12* | *Tnfrsf13b* |
| *SNP2* | *rs26970024* | SNP F | 11:61091054 | *Aldh3a2* | *Slc47a2* |
| *SNP3* | *rs26941299* | SNP E | 11:61265795 | *Rnf112:* Exon | *Rnf112:* Exon |
| *SNP4* | *rs26955056* | SNP A | 11:61679215 | *A530017D24Rik:Exon* | *A530017D24Rik:Exon* |
| *SNP5* | *rs26954885* | SNP G1 | 11:61739238 | *Akap10:* Intron | *Akap10:* Intron |
| *SNP6* | *rs26941842*,  *rs26941841*,  none,  *rs26941840* | SNP B | 11:61862912,  11:61862942,  11:61862991,  11:61863082, | *Specc1: Intron* | *Specc1: Intron* |

Official designations are from dbSNP Build 132. Base-pair positions on mouse Chromosome 11 are from NCBI Build 37. These data accessed through the Mouse Genome Database at the Mouse Genome Informatics website, The Jackson Laboratory, Bar Harbor Maine. World Wide Web (URL: <http://www.informatics.jax.org> (Accessed September, 2012) and the ensembl Mouse Genome Database, release 64 <http://ensembl.org/Mus_musculus> (Accessed September, 2012).
