# Supplementary material for: The wooly mutation (wly) on mouse chromosome 11 is associated with a genetic defect in Fam83g
Source: BMC Res Notes. 2013 May 9;6:189. doi: 10.1186/1756-0500-6-189 (PMC3663780; doi:10.1186/1756-0500-6-189)
Supplement: Additional file 3 — DNA re-sequencing analysis of the exonic portions of the overlapping Slc5a10 and Fam83g genes in mouse. [file 1756-0500-6-189-S3.docx]

9

*

*

*

*

*

*

‡ * *

*

*

*

8

*

*

***Fam83g >***

3

5

4

1

*

†

**

*

1

3

4

5

6

7

11

12

*

*

*

*

*

*

†

†

‡

‡

*

*

*

*

*

***

*

‡

‡

*

*

*

*

13

15

16

14

10

2

‡

*

*

†

2

***< Slc5a10***

**Additional file 3.** DNA re-sequencing analysis of the exonic portions of the overlapping *Slc5a10* and *Fam83g* genes in mouse. Taller boxes represent coding regions, shorter boxes represent 5’ or 3’ untranslated regions (UTRs). *Slc5a10* is transcribed from the reverse strand, while *Fam83g* is transcribed from the forward strand. Exon 5 of *Fam83g* includes Exons 7, 8, and 9 of *Slc5a10*; Exon 10 of *Slc5a10* is within Intron 4-5 of *Fam83g*; Exons 1 to 4 of *Fam83g* are included in Intron 10-11 of *Slc5a10*. Exons shown in green (and ten to a few hundred flanking bases) were amplified from A/J, C57BL/6J, NOD/ShiLtJ, and NOD/ShiLtJ-*wly*/J DNA templates by a standard PCR reaction; and these amplimers were sequenced by primer-extension analysis (SeqWright, Inc.; Houston, TX, USA). Asterisks (*) denote single nucleotide polymorphisms (SNPs) between A/J and C57BL/6J versus the NOD strains which have previously been identified and assigned re-sequencing designations in dbSNP, Build 132. (The blue asterisk denotes the same polymorphism in both genes.) Daggers (†) denote small (1 to 5 bp) intronic deletions in the NOD strains but not in A/J or C57BL/6J which have not been previously described. Double daggers (‡) denote polymorphisms not previously described between the NOD strains and the standard A/J and C57BL6J strains. These *Slc5a10* and *Fam83g* polymorphisms are described in detail in **Additional files 4 & 5**. The portion of Intron 2-3 and the 5’ end of Exon 3 in *Fam83g* shown in red (including 4 SNPs represented by red asterisks) is deleted in NON/ShiLtJ-*wly*/J only. This *wly*-specific deletion is described in **Additional file 6a**.
