# Supplementary material for: The wooly mutation (wly) on mouse chromosome 11 is associated with a genetic defect in Fam83g
Source: BMC Res Notes. 2013 May 9;6:189. doi: 10.1186/1756-0500-6-189 (PMC3663780; doi:10.1186/1756-0500-6-189)
Supplement: Additional file 4 — Description of polymorphisms encountered while re-sequencing Slc5a10 in A/J, C57BL/6J, NOD/ShiLtJ, and NOD/ShiLt-wly/J mouse DNA. [file 1756-0500-6-189-S4.docx]

**Additional file 4.** Description of polymorphisms encountered while re-sequencing *Slc5a10* in A/J, C57BL/6J, NOD/ShiLtJ, and NOD/ShiLtJ-*wly*/J mouse DNA.

| **Location in *Slc5a10* (Type)** | **Designation** | **Map Position (NCBI Build 37)** | **Alleles (Strains A/B/N/W)** | **Sequence** |
| --- | --- | --- | --- | --- |
| Exon 3 (Silent) | *rs50666083* | 11:61,529,771 | G/G/A/A | 5' cagTCGGCTT GTCGAGCCAA CAAGAACACA GTGAGCGGCT ACTTCCTGGC  **R**  GGCCGGGACA TGGCTTGGTG GCCGgtgagt tcacccacct tctagcctct 3' |
| Intron 4-5 | *ΔI4-5* | 11:61,528,933-7 | tgtgg/tgtgg/•••••/••••• | 5’ tccgtaagaa gctcctagct tctcacagca aagcaggagg ggcgtgtgtg  **Δ**  ggggggggta gcctcagcac tactcctctc tgccacatct ggctgtgtgc 3’ |
| Intron 7-8 | *rs269696673* | 11:61,522,894 | g/g/a/a | 5’ aaggaatgag gccccaccag cagctctggt gatgaggtca gactctcctg  **r**  gacacttact ccagcagcct tgtttcttgc agGGGGCCTG GCCACTGTGA 3’ |
| Intron 9-10 | *rs26969694*,  *rs26969695* | 11:61,518,038;  11:61,517,921 | a/a/g/g,  c/c/t/t | 5’ ggagtaggag actgcttcct tggggtaagt gtagcagaag ggagagcgcc **r**tgggatgtc agctgtgcgt gctggtcatc ttaggtggta tagggataac aacactccat ctaccttgtg tcacttcatg aggcccatga aggaggttga tactggtatc cccaagc**y**  gacagtcttc atctcttttc tcctgtgccc agGTTATTGT GCAGCGGTCC 3’ |
| Intron 11-12 | *rs50378466,*  *rs50695289,*  *rs51780222,*  *rs49262176,*  *rs50864461,*  *rs46197350,*  *rs50277952* | 11:61,492,015; 11:61,492,008; 11:61,492,005; 11:61,491,984; 11:61,491,952; 11:61,491,949; 11:61,491,914 | g/g/a/a,  a/a/g/g,  g/g/t/t,  c/c/t/t,  c/c/a/a,  g/g/a/a,  a/a/g/g | 5’ AGCTGATGCC CATAGgtgag tgactatctc atggacagat gaaaggtcct  **r**gtgagt**r**ag **k**gttggagct gtggggtcca c**y**ctgcatcc tgcacgagtc cctgtgccat  cca**m**tc**r**gca cagacagaag tgcccagaag taaaggccca g**r**  cttcccacgt cagctctttc tccttcctca gactgcttgg ctgggctctt 3’ |
| Intron 11-12 | *rs49683355* | 11:61,489,907 | g/g/a/a | 5’ tgagagcagc ccaagcactg ggtaattact tccaggctcc cggctccacc  **r**  ccctttcaga agtctcccac agccctgcca ctggctgccc cttgagtcct 3’ |
| Intron 12-13 | *ΔI12-13* | 11:61,487,560 | t/t/•/• | 5’ cgtggttctt agtggcctgt gctgaaatgt gcaaaggggt ccagaggcag  **Δ**  ttttttttgg gggggggggc agtgaagaac tgaggcaggg ctggcctctg 3’ |
| Intron 13-14,  Exon 14 (Silent) | *rs51936858,*  *SNP A* | 11:61,487,249;  11:61,487,220 | c/c/t/t,  T/T/C/C | 5’ ccttaagggt actccgcaag tcctttgaga **tcttcctctg tctctccttt**  **y**cccagGGGG CCTTCTGGGG CCTGATGGC**Y**  GGGCTGGTGG TGGGTGCTCT GAGGCTGGTC CTGGAATTCC TGTACCCGGA 3’ |
| Intron 14-15 | *rs47529221,*  *rs46778988,*  *rs49730561* | 11:61,486,905;  11:61,486,871;  11:61,486,834 | g/g/a/a,  a/a/g/g,  c/c/g/g | 5’ ctctgttcac atccagatct ctgccccttt atgatgccag gtacccccac  **r**gttgactcc taaaccttgc tggcccagac cttc**r**tcttt gtttgaacta agcttccttc  tttaactgac c**s**  gcatcttctc cggggctgct ctctacacct ctcacgccct ttctctgcag 3’ |
| Intron 15-16,  Exon 16 (3’ UTR),  Downstream | *rs51689839,*  *rs50111507,*  *rs46437353,*  *SNPB* | 11:61,486,630;  11:61,486,629;  11:61,486,477;  11:61,486,282; | a/a/t/t,  t/t/c/c,  C/C/T/T,  c/c/t/t | 5’ gggctggagg tatggggact gaaagtatag ccttcatccc tcactggcct  **wy**ttcaccct cctctagGTG ATGGCCAAAC ACCCCAGAAA CGTGCTTTCT GGGCCCGCGT GTGTAATGTC AACGCCATCT TCCTCATGTG TGTCAACATT TTCTTCTATG CCTATTTTGC CTGATGCTGC CACCACACCA GTGGGAAGAC AGG**Y**GCTTCA AGTTCTCAGG ACCACCTTCC TTCCTGGGTT GGACATGAAG GCCTAAGGAA TAGAATGTGC CCACAGATAA ACAGTGGTCA ATACCAAGTG TCTGGCTGAG CCAGCAGACA CGGTGCTCTG AAAATATAGT GGAGAATCAT  TTCAAAAATA GCACTTTAAA CAAAGTCAGA TATGGGGAGA AAGATTGa**y**  gtccactggt taatgtttta ttttgaatac gcaggtacag gttaggagca 3’ |

DNA from strains A/J (abbreviated A), C57BL/6J (abbreviated B), NOD/ShiLtJ (abbreviated N), and NOD/ShiLtJ-*wly*/J (designated W) was sequenced by primer extension (SeqWright, Inc.; Houston Texas). The official “*rs#*” designations shown are from dbSNP Build 132, other designations are informal. Base-pair positions on mouse Chromosome 11 are from NCBI Build 37, and the reverse strand is shown. These data were accessed through the Mouse Genome Database (MGD) at the Mouse Genome Informatics website, The Jackson Laboratory, Bar Harbor Maine. World Wide Web (URL: <http://www.informatics.jax.org> (Accessed September, 2012). Sequences shown in black capitals are exonic; purple upper-case sequences are from the UTR, blue lower-case sequences are intronic green lower-case sequences are downstream of the gene. Nucleotides that differ between two strains are shown in red, where M = A or C, S = C or G, K = G or T, R = A or G, W = A or T, and Y = C or T; Δ designates a deletion of base(s), as indicated.
