# Supplementary material for: The wooly mutation (wly) on mouse chromosome 11 is associated with a genetic defect in Fam83g
Source: BMC Res Notes. 2013 May 9;6:189. doi: 10.1186/1756-0500-6-189 (PMC3663780; doi:10.1186/1756-0500-6-189)
Supplement: Additional file 5 — Description of polymorphisms encountered while re-sequencing Fam83g in A/J, C57BL/6J, NOD/ShiLtJ, and NOD/ShiLt-wly/J mouse DNA. [file 1756-0500-6-189-S5.docx]

**Additional file 5.** Description of polymorphisms encountered while re-sequencing *Fam83g* in A/J, C57BL/6, NOD/ShiLtJ, and NOD/ShiLtJ-*wly*/J mouse DNA.

| **Location in *Fam83g* (Type)** | **Designation** | **Map Position (NCBI Build 37)** | **Alleles**  **(Strains A/B/N/W)** | **Sequence** |
| --- | --- | --- | --- | --- |
| Intron 1-2 | *rs51454780,*  *rs49574701* | 11:61,498,623;  11:61,498,675 | t/t/a/a,  t/t/c/c | 5' gagaccccca ttcacccagc acgcatgggc accgagacag cccctggacc  **w**ctgactggc ctctcggttg cctcctccgg tgatcagaag ctgaatgtcc ac**y**  ccctctagca ggtggcactg ggcttccagg ctgagtgggc ttctgtgccc 3' |
| Intron 1-2,  Exon 2 (Silent),  Intron 2-3 | *rs26969728,*  *rs26969727,*  *rs48632639* | 11:61,508,601;  11:61,508,626;  11:61,508,810 | c/c/t/t,  C/C/A/A,  c/c/g/g | 5' ggagcaaggg tgctgtcctc attgctgtgc tggtaaacca cccagt**ctttt**  **y**ctgttccca gGTGATAGCT GTGGT**M**ATGG ACATGTTCAC TGACGTGGAC ATCTTCAAGG ACCTGCTGGA TGCTGGCTTC AAGAGGAAGG TGGCTGTGTA CATCATTGTG GATGAGAGTA ATGTCAAGTA TTTCCTGCAC ATGTGTGAGC GGGCCCGCAT GCACCTGGGG CACCTCAAGg tgagctgtcg tcccctgctt gggcagctg**s**  agggcaggta gtcatttagt cagtcaacaa acattgcctt gcagacagtg 3' |
| Intron 2-3 | *rs26969702* | 11:61,514,416 | a/a/g/* | 5' aacctggaac cactgagtgg gagagagagc agacgggcaa gtcacggctc  **r**  gtctgacaaa gactttgtca ccaacctgtg tttgaatcca gagctcccac 3' |
| Intron 2-3 | *rs48301379,*  *rs51773890* | 11:61,514,596;  11:61,514,599 | c/c/t/*,  t/t/c/* | 5' tgttctttgg gagcctcagc agtccctttc tgcttagatt cactcagact  **y**cc**y**  ttgcccctgc gttccctgtg ctcagcaatg ggtcctcaag cacccaggct 3' |
| Intron 2-3 | *rs26969701* | 11:61,515,062 | t/t/c/* | 5' ttctccacct tgcacctcac tcccggtcct caatgcctac acactgcctg  **y**  gggctctgga cagtctgatg tccctgcctg tgaagtcagt gtctgtagtt 3' |
| Intron 3-4 | *rs26969699*  *SNP C* | 11:61,515,808,  11:61,515,855 | a/a/g/g,  a/a/t/t | 5' tccagactca ggtttgaccc ctgtcccacc ccatgccacc tagtctgata  **r**accccaggt agtgcactgt tgatgccccc tatctcaggc caggggg**w**  gtatgcactg ttgatgccca ctgcctcggg ccaggggcac tcactgaatc 3' |
| Exon 4 (Silent) | *rs26969698* | 11:61,516,088 | C/C/T/T | 5' CAGTTCCAGG AGCTGTACCT TATGTCTCAG AGTGTCAGCC TCAAGGACAT  **Y**  CCCATGGAGA AGGAGCCAGA GCCAGAGCCC ATTGTGCTAC CATCTGTGGT 3' |
| Exon 4 (Non-synonymous),  Exon 4 (Non-synonymous) | *rs26969697,*  *SNP D* | 11:61,516,696;  11:61,516,774 | G/G/A/A,  C/C/T/T | 5' GGCTCCTGTA CCTAAGCCCC GGACAGTCCC TGTGGCAAGT GTACTTGCCC  **R**GGATGGTAG TGATATTGGC TGGGCCCTGG ATACCCCAGA AAAGGAGACA CCCCAAAATG GGATAGACCC CAGGCTAC**Y**  AAGCACAGCC AGCGAAAGCG AGGTCCCACA ACAGCAGCAC TCATCTATGA 3' |
| Exon 5 (Silent) | *rs26969679* | 11:61, 520,893 | T/T/C/C | 5' actcactctc ctttttctct ccagGGCCAG CATTTCCACC GACATGGAAG  **Y**  ACTACCTCAA GGACCCCAGG GCCTCCCCGT TTCCGGCCTG CTGCTGATGG 3' |
| Exon 5 (3’ UTR) | *SNP E,*  *ΔE5a,*  *rs47344066* | 11:61,521,647-8;  11:61,521,653-4;  11:61,521,738 | GA/GA/AG/AG,  GA/GA/••/••,  C/C/T/T | 5' AACCCCTACC TCATATTTCC TGCTCCAGGA AGGTTGAGGT TGAAAAAAAA  **RR**AAAA**ΔΔ**GT GAAATAGAGT ATTTTATTTG TATTTAAAAT AACTATTAAA TGAGCATTTT GCACATGGGC CCACAGTGTC CTGTTGGCCA G**Y**  GGGGTTCACG GCTAGTGTCT GGAAGCCTGC TGGCCTCGGG TATGGGCTTC 3' |
| Exon 5 (3’ UTR) | *rs26969676* | 11:61,521,986 | C/C/T/T | 5' CCAAGACACA GGGAAGGAAG GCAGAGGCTC CTCAGGGCCT TATGGTAGGC  **Y**  TAGCAGGTAG AGCTCAGGCC AGGGGTCTTT CCTCTGAGTC GGGAACATGT 3' |
| Exon 5 (3’ UTR) | *rs26969674* | 11:61,522,111 | T/T/C/C | 5' TGCGAGGCCA AGCCAGGCCT GGCTGTGTCT GTAGTCATTT GCCCTCTCAT  **Y**  CGGCCTATAT CTGACCATTA GGTCCCTCCC CTTCAGTAGG TCCTGGAGAC 3' |
| Exon 5 (3’ UTR) | *SNP F,*  *ΔE5b rs47321118* | 11:61,522,240;  11:61,522,286-7;  11:61,522,299 | C/C/T/T,  TC/TC/••/••,  T/T/A/A | 5' ACCTGGGTGT GGGGCAGCTT CTGGCATCTT CCCAGGCCCC CTTTCCCAGC  **Y**GGGTGTGGA GCTAGGTAGG CTAGGGTCTG TTTCACCAGG AATCTC**ΔΔ**CT GAGGCCCAG**W**  GGCAGCCTCT GTCACAGGGT CCCAGAGCCT GAGCTGAGGC GTCACCCTTA 3' |
| Exon 5 (3’ UTR) | *rs45632976* | 11:61,522,594 | G/G/A/A | 5' AACACCTCAG CAGTTTTGTG GTCTGCTAAT TGTGTGCTGC ACACACAGCT  **R**  CCTGCTCTAG GGCCTGCCCA GCCCTGAGGG AGGCTAGTCA GCGCCTCCCT 3' |
| Exon 5 (3’UTR) | *rs26969673* | 11:61,522,894 | C/C/T/T | 5' TCACAGTGGC CAGGCCCCCT GCAAGAAACA AGGCTGCTGG AGTAAGTGTC  **Y**  CAGGAGAGTC TGACCTCATC ACCAGAGCTG CTGGTGGGGC CTCATTCCTT 3' |

DNA from strains A/J (abbreviated A), C57BL/6J (abbreviated B), NOD/ShiLtJ (abbreviated N), and NOD/ShiLtJ-*wly*/J (designated W) was sequenced by primer extension (SeqWright, Inc.; Houston Texas). The official “*rs#*” designations shown are from dbSNP Build 132, other designations are informal. Base-pair positions on mouse Chromosome 11 are from NCBI Build 37. These data were accessed through the Mouse Genome Database at the Mouse Genome Informatics website, The Jackson Laboratory, Bar Harbor Maine. World Wide Web (URL: <http://www.informatics.jax.org> (Accessed September, 2012). Sequences shown in capitals are exonic (black are coding regions, purple are untranslated regions); blue lower-case sequences are intronic. Nucleotides that differ between two strains are shown in red, where M = A or C, S = C or G, R = A or G, W = T or A, and Y = C or T; Δ designates a deletion of bases, as indicated. A red asterisk indicates that the polymorphism lies within the 955 bp *wly*-specific deletion shown in **Additional file 6a** of Radden *et al.* (2013).
