# Supplementary material for: The wooly mutation (wly) on mouse chromosome 11 is associated with a genetic defect in Fam83g
Source: BMC Res Notes. 2013 May 9;6:189. doi: 10.1186/1756-0500-6-189 (PMC3663780; doi:10.1186/1756-0500-6-189)
Supplement: Additional file 6 — A Fam83g deletion specific to the NOD/ShiLtJ-wly/J strain alters mRNA splicing. [file 1756-0500-6-189-S6.docx]

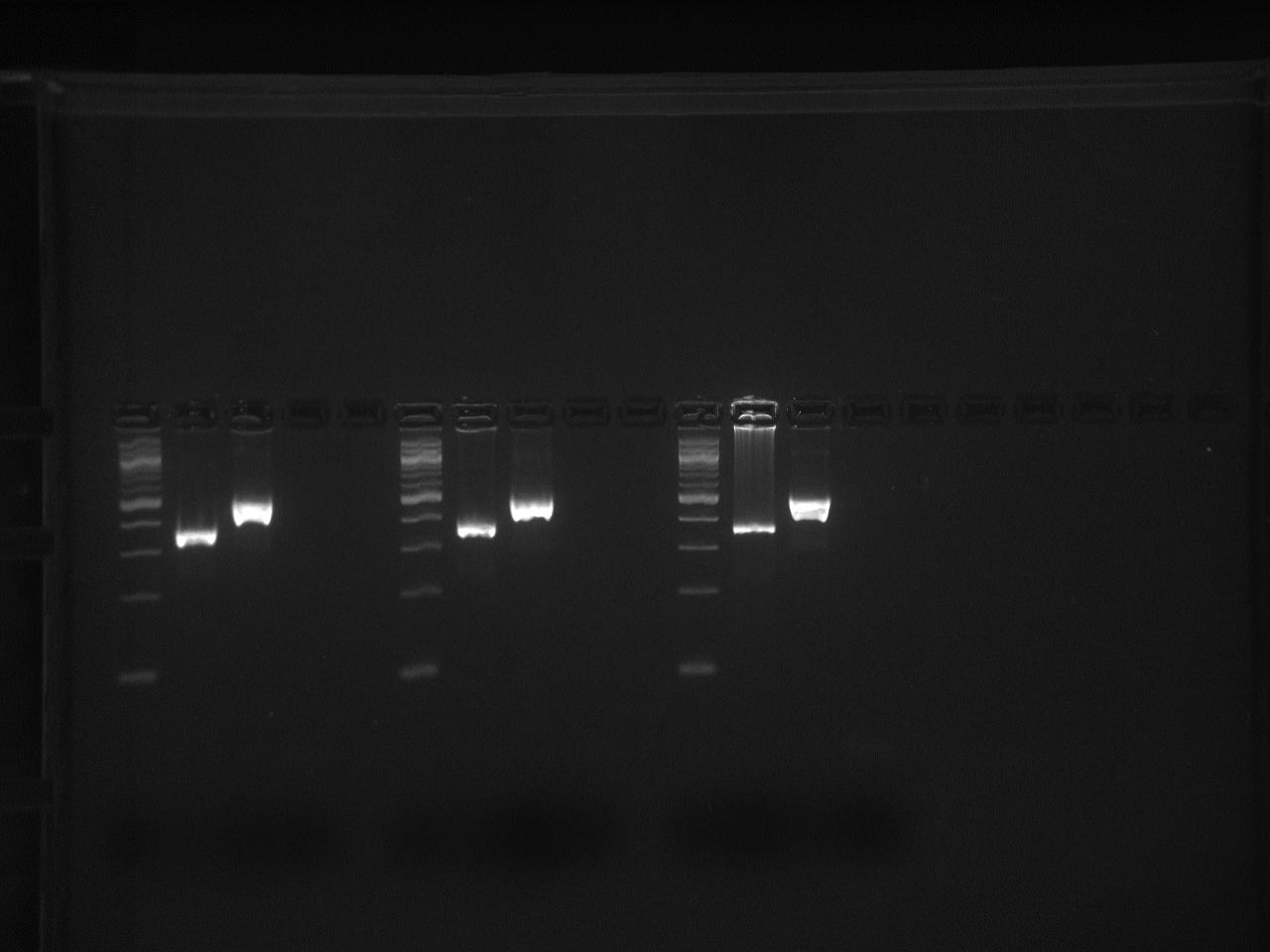

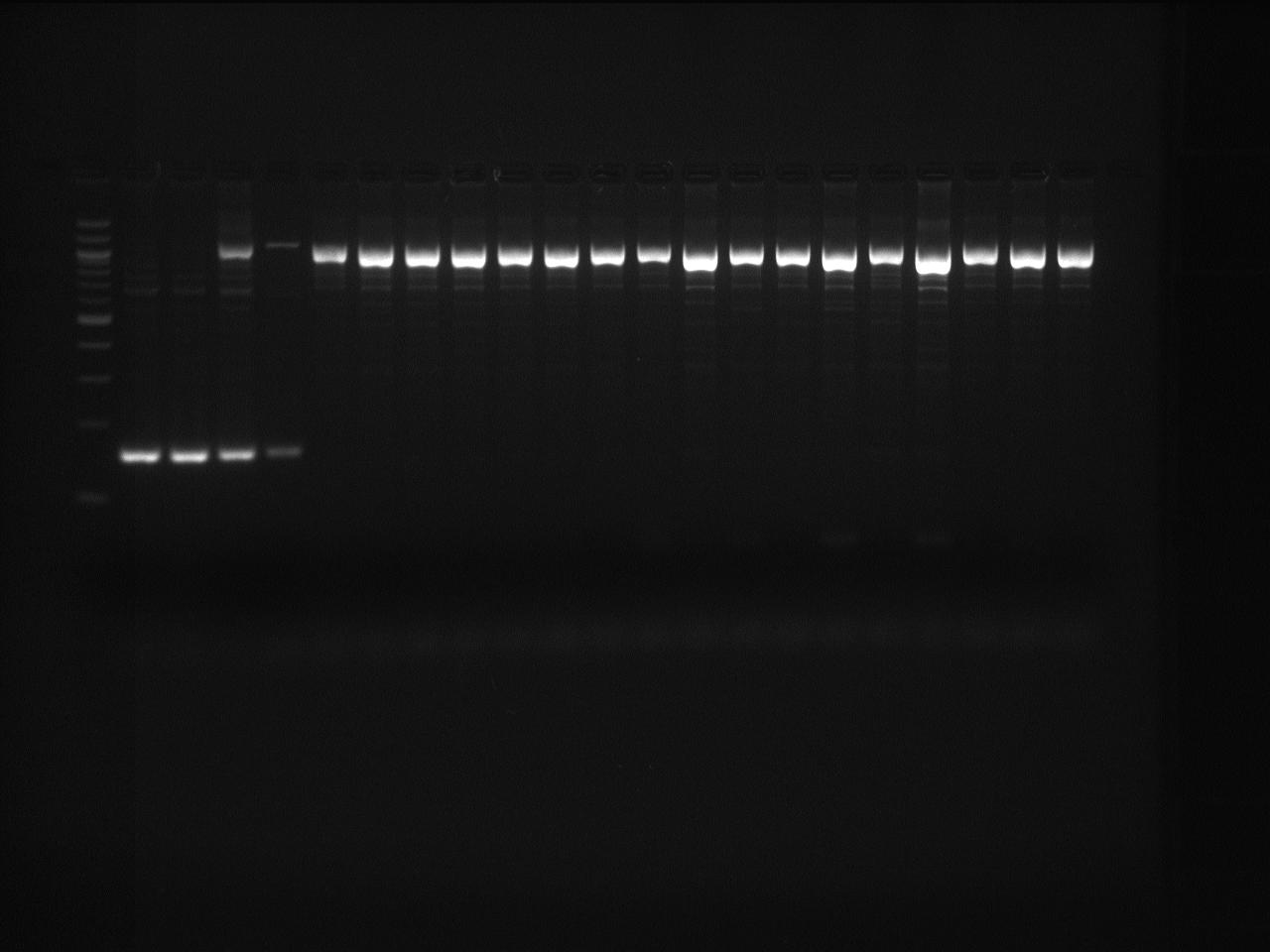

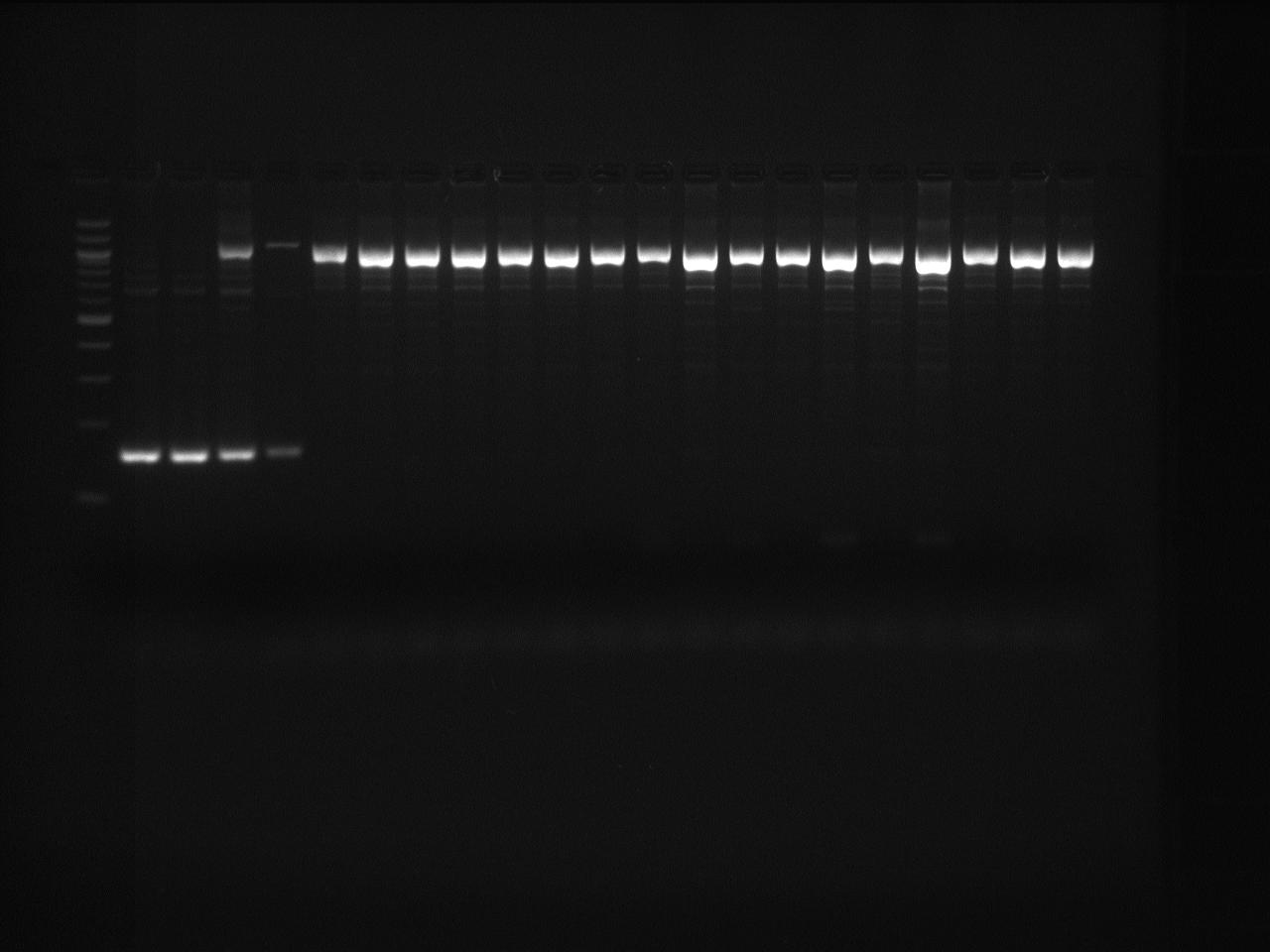


**5’attcactcctctgcttctgggcattccaggctgttgttttgactcactgccacctccaca 61514281**

**gtagcaccttgtcactgccatctttgtctgcctctgggggcctcagtaggggtcagtgaa 61514341**

**caaacaagcatgtgggcagttagtaacctggaaccactgagtgggagagagagcagacgg 61514401**

**gcaagtcacggctcrgtctgacaaagactttgtcaccaacctgtgtttgaatccagagct 61514461**

**cccaccatgtgagcttcatgtaagtagaccagtcaggaagctctgctgagccttaagcca 61514521**

**gatggaccctaggtccctacctactgttctttgggagcctcagcagtccctttctgctta 61514581**

**gattcactcagactyccyttgcccctgcgttccctgtgctcagcaatgggtcctcaagca 61514641**

**cccaggctctggatggttctagaggcactgcactgaagagaaaggccagcagtttgtggc 61514701**

**cccactttctctctaaacctctgcaagctcttctgggctctgagggctttcccctgctgg 61514761**

**ctctaagtcaggctgtgtggtagaagctgaagttgactatgagattgtcccagaccatgc 61514821**

**cccatctccagaggaagagttttctgagtactttcaagggagcctgcttagcagttcagt 61514881**

**gacatgggatgcaagactggggtgaggcctgtgatcagtcatcctggaagaccctggatg 61514941**

**tgttaaagtgtaagacccaggttccatcctccttgcatggccccacagtccagatacact 61515001**

**accactacccttctccaccttgcacctcactcccggtcctcaatgcctacacactgcctg 61515061**

**ygggctctggacagtctgatgtccctgcctgtgaagtcagtgtctgtagttgtctaattc 61515121**

**tgcctccctgtctcccaacacagAATCTCAGGGTACGCAGCAGCGGGGGAACCGAGTTCT 61515181**

**TCACACGATCAGCCACCAAGTTCAAGGGTGTCCTGGCCCAGAAGTTCATGTTTGTGGATG 61515241**

**GAGATCGGGCCGTGTGTGGCTCCTACAGgtgattctcctctgcagcttggggaggagttg 61515301**

**cctctggtttgcagctggtttctgccctta 3’ 61515331**

**(c)**

**NOD/ShiLtJ-*wly*/J**

**NOD/ShiLt/J**

**100 bp ladder**

**NOD/ShiLtJ-*Lepr^db-5J^*/J**

**NOD/ShiLtJ-*Npr3^lgj-4J^*/J**

**SWR/J**

**NOD/ShiLt/J**

**(NOD-*wly* x A/J)N_2_**

**NOD/ShiLt**

**NOD/ShiLtJ-*wly*/J**

**NOD/ShiLtJ-*wly***

**C57BL/6J**

**BALB/cByJ**

**ICR/HaJ**

**100 bp ladder**

**NON/ShiLtJ**

**SJL/J**

**DBA/2J**

**FS/EiJ**

**C3H/HeJ**

**BKS(Cg)-*frzl*/J**

**AKR**

**A/J**

**NOR/LtJ**

**← wild**

**← mutant**

**← wild**

**(b)**

**(a)**

**← mutant**

**Additional file 6.** A *Fam83g* deletion specific to the NOD/ShiLtJ-*wly*/J strain alters mRNA splicing. (a) DNA sequence that defines the *wly*-specific *Fam83g* deletion. Sequence from *Fam83g*, Intron 2-3 and 3-4 is shown in lower-case; sequence shown in upper-case is from Exon 3. Sequence shown in red is deleted in NOD/ShiLtJ*-wly*/J mice. Bases highlighted in yellow are polymorphic among the 4 strains sequenced here, and are described in detail in **Additional file 5**. (b) The 955 bp *Fam83g* deletion is found only in *wly* carriers and homozygotes. Shown are PCR products amplified from genomic DNA samples isolated from the various strains indicated above each lane. The reaction was directed by primers (underlined bases in Part a) that flank the deletion. The panel of mice tested included 19 inbred strains and 2 hybrids, including 6 distinct NOD strains; 1 strain derived from NOD (NOR); 4 strains of Swiss ancestry related to NOD (ICR, SJL, SWR, and NON); and 8 additional strains (not closely related to NOD). (c) PCR analysis of cDNA templates reveals altered splicing of the mutant *Fam83g* transcript. Total RNA isolated from mutant NOD/ShiLtJ*-wly*/J or wild type NOD/ShiLtJ skin was copied into cDNA, and amplified using primer sequences located in Exons 1 and 4. While amplimers copied from the mutant cDNA measured 359 bp (and were shown by sequence analysis to lack Exon 3), those copied from the wild type cDNA measured 484 bp (and included Exon 3, as expected).
